# Exploring the Mechanism of the miRNA-145/Paxillin Axis in Cell Metabolism During VEGF-A–Induced Corneal Angiogenesis

**DOI:** 10.1167/iovs.62.10.25

**Published:** 2021-08-23

**Authors:** Wanju Yang, Yanning Yang, Shanshan Wan, Ya Xu, Jing Li, Lu Zhang, Wanruo Guo, Yijun Zheng, Yi Xiang, Yiqiao Xing

**Affiliations:** 1 Aier Eye Hospital of Wuhan University, Wuhan, China; 2 Department of Ophthalmology, Central Hospital of Wuhan, Tongji Medical College of Huazhong University of Science and Technology, Wuhan, China; 3 Eye Center, Renmin Hospital of Wuhan University, Wuhan, China

**Keywords:** paxillin, angiogenesis, endothelial cells, vascular endothelial growth factor

## Abstract

**Purpose:**

Paxillin (PXN) is a key component of focal adhesions and plays an important role in angiogenesis. The aim of the present study was to investigate the effect of PXN in vascular endothelial growth factor A (VEGF-A)–induced angiogenesis in human umbilical vein endothelial cells (HUVECs).

**Methods:**

HUVECs were transfected with PXN overexpression and PXN interference vectors. Biochemical detection was used to detect adenosine triphosphate and lactic acid production. The morphology of mitochondria was observed under an electron microscope, and flow cytometry was conducted to measure mitochondrial membrane potential. Transwell experiments were used to detect the migration and tube formation ability of each group of cells. The expression of hexokinase (HK)1, HK2, glucose transporter 1 (GLUT1), phosphorylated phosphatidylinositol 3-kinase (PI3K), phosphorylated AKT, and phosphorylated mechanistic target of rapamycin (mTOR) was evaluated by western blot.

**Results:**

PXN silencing reduced the levels of lactic acid and adenosine triphosphate, downregulated HK1, HK2, and GLUT1, suppressed PI3K/AKT/mTOR signaling activation, and inhibited VEGF-A–induced mitochondria injury in VEGF-A–induced HUVECs. We also determined that miR-145-5p decreased the VEGF-A–induced expression of PXN and inhibited the invasion and angiogenesis of HUVECs. Also, miR-145-5p inhibition blocked the protective effect of PXN interference on VEGF-A–induced HUVEC injury. Furthermore, PXN interference significantly decreased lactic acid and adenosine triphosphate levels, inhibited PI3K/AKT/mTOR activation, and decreased the levels of HK1, HK2, and GLUT1 in VEGF-A-treated mouse corneal.

**Conclusions:**

The results indicate that PXN silencing inhibited the VEGF-A–induced invasion and angiogenesis of HUVECs via regulation of cell metabolism and mitochondrial damage, suggesting that PXN may be a potential target for antiangiogenic therapies.

Paxillin (PXN) is the main component of focal adhesions and plays an important role in the transduction of extracellular signals into intracellular responses, which is triggered by the engagement of integrins with the extracellular matrix.^1^ PXN is related to cancer cell movement, migration, and invasion and ultimately shows a certain correlation with tumor metastasis. Studies have shown that functional mutations in PXN are associated with the malignant progression of many tumors.^2^ Cellular processes such as angiogenesis, proliferation, self-renewal, and differentiation require a large amount of energy and materials. Studies have shown that the expression of PXN can affect mitochondrial kinetics and that *PTEN* can inhibit the transcription of PXN through phosphatidylinositol 3-kinase (PI3K)/AKT/nuclear factor kappa B signaling, thereby inhibiting tumorigenesis and development.^3^^,^^4^

PI3K/AKT/mechanistic target of rapamycin (mTOR) is the core signaling pathway of cellular energy metabolism and plays an important role in angiogenesis. The activation of PI3K might be induced by a *RAS* mutation, which upregulates the expression of growth factor receptors, such as epidermal growth factor receptor, or downregulates *PTEN*.^5^ It was reported that vascular endothelial growth factor (VEGF) secretion can be induced by the activation of PI3K/AKT/mTOR signaling.^6^ Furthermore, PI3K/AKT signaling regulates the levels of other angiogenic factors, including angiopoietins and nitric oxide.^7^

By directly targeting key secretory factors and transcription factors, miRs exert powerful effects in controlling angiogenesis in both cell-autonomous and non–cell-autonomous ways.^8^ As a tumor suppressor, miR-145 has been reported to be expressed at low levels in tumor cells and has inhibitory effects against tumor growth, migration, and invasion.^9^ Additionally, miR-145 was found to play a negative regulatory role in the proliferation and migration of vascular smooth muscle cells.^10^ Furthermore, miR-145 is expressed in vascular endothelial cells. Climent et al.^11^ found that miR-145 overexpression in endothelial cells reduced their ability to form capillary-like structures. Studies have found that miR-145 mediates mitogen-activated protein kinase and PI3K/AKT signaling in tumor cells to inhibit tumor proliferation, migration, and invasion.^12^^,^^13^ However, whether miR-145 affects cellular metabolism through PI3K/AKT signaling has not been investigated.

This study revealed that VEGF-A induces significant differential expression of intracellular PXN, which in turn affects cell migration. At the same time, it further affects cell adhesion and angiogenesis by participating in intracellular energy and material metabolism. On the other hand, the expression of PXN is regulated by miR-145. Under inflammatory conditions, the expression of miR-145 is significantly downregulated, which leads to the overexpression of PXN, further promoting angiogenesis and cell growth in an inflammatory environment.

## Materials and Methods

### Cell Lines and Culture Conditions

Human umbilical vein endothelial cells (HUVECs) were purchased from the cell resource center of the Shanghai Biological Sciences Institute (Chinese Academy of Sciences, Shanghai, China). Cells were cultured in special medium for HUVECs (PromoCell, Heidelberg, Germany) supplemented with 20% fetal bovine serum (FBS; Gibco, Waltham, MA, USA) at 37°C in a humidified atmosphere of 5% CO_2_. For VEGF-A treatment, HUVECs were pre-incubated with 20 ng/mL VEGF-A for 1 hour.

### Cell Transfection

PXN overexpression vectors (pLVX-PXN) and interference vectors (pSICOR-shPXN) were obtained from Wuhan Myhalic Biotechnological Co., Ltd. (Wuhan, China) and transfected with Invitrogen Lipofectamine 2000 (Thermo Fisher Scientific, Waltham, MA, USA) according to the manufacturer's guidelines. The sequences for PXN overexpression were forward: GGAATTCATGGACGACCTCGACGCCCTG, and reverse: CTCTAGACTAGCAGAAGAGCTTGAGGAA. The sequence for shPXN was GGGCAGCAACCTTTCTGAACT. After 24 hours, the transfected cells were harvested for total protein extraction. The efficiency of transfection was confirmed by western blot. For overexpression and inhibition of miR-145-5p, mimics and inhibitors were used, respectively. Mimics and inhibitors were acquired from RiboBio (Guangzhou, China) and administered at 50 nM.

### Quantitative RT-PCR

The total RNA was extracted using TRIzol (Invitrogen), and the cDNA was obtained by reverse transcription RNA (Takara Bio, Kyoto, Japan). The reaction system was prepared according to the manufacturer's instructions. *U6* was used as the standard internal reference for mir-145-5p, and the primer was synthesized by Shanghai Shenggong Biological Engineering Technology Service (Shanghai, China). After 30 seconds of pre-denaturation at 94°C, 5 seconds at 94°C, annealing at 60°C for 15 seconds, and 72°C for 10 seconds, 45 cycles were amplified. Quantitative analysis was performed using the comparative threshold method, and the calculation method was as follows: quantitative copy number of target gene = 2^–ΔΔ^^CT^, where ΔΔCT = ΔCT experimental group – ΔCT control group. The copy number of the target gene was calculated for each specimen.

### Western Blot

Cells were washed twice with PBS and homogenized in radioimmunoprecipitation assay lysis buffer (Beyotime Biotech, Suzhou, China) containing protease inhibitors at 4°C and centrifuged at 12,000*g* for 15 minutes. Then, 30 µg of proteins were separated by 10% sodium dodecyl sulfate–polyacrylamide gel (SDS-PAGE) and transferred onto polyvinylidene fluoride membranes (MilliporeSigma, Burlington, MA, USA). The membranes were blocked with 5% skim milk for 2 hours at room temperature in Tris-buffered saline. The membranes were then incubated overnight at 4°C with primary antibodies against PXN (Bioswamp, Wuhan, China): (molecular weight 102 kD PAB36563, 1:1000); hexokinase (HK)1 (120 kD, PAB30519, 1:1000); HK2 (102 kD, PAB30271, 1:1000); glucose transporter 1 (GLUT1; 54 kD, PAB34949, 1:1000), PI3K (124 kD, PAB30084, 1:1000), phospho-PI3K (84 kD, phosphorylation sites Y467/Y199/Y464, PAB4641-P, 1:1000), AKT (60 kD, PAB30596, 1:1000), phospho-AKT (56 kD, phosphorylation site S473, PAB4318-P, 1:1000), mTOR (290 kD, PAB30674, 1:1000), and phospho-mTOR (289 kD, phosphorylation site S2448, PAB36313-P, 1:1000). Glyceraldehyde-3-phosphate dehydrogenase (36 kD, PAB36269, 1:1000; Bioswamp) was selected as the internal reference.

The membranes were washed with Tris-buffered saline and incubated in biotinylated goat immunoglobulin G secondary antibody (SAB43714, 1:20000; Bioswamp) for 2 hours at room temperature. Immunoreactivity was visualized by colorimetric reaction using an enhanced chemiluminescence substrate buffer (MilliporeSigma). Membranes were scanned with a Gel Doc EZ imager (Bio-Rad Laboratories, Hercules, CA, USA).

### Lactic Acid and Adenosine Triphosphate Detection

The levels of lactic acid and adenosine triphosphate (ATP) in HUVECs were detected by enzyme-linked immunosorbent assay (ELISA) kits (A019-2 and A095, respectively; Nanjing Jiancheng Bioengineering Institute, Nanjing, China) according to the manufacturer's guidelines.

### Transmission Electron Microscopy

For morphological observation, HUVECs were fixed with 4% paraformaldehyde, fixed again in 1% osmium tetroxide, dehydrated in graded ethanol, and embedded in Epon. The sections were placed on formvar/carbon-coated, 200-mesh copper electron microscopy grids, incubated for 5 minutes at room temperature, and subjected to standard uranyl acetate staining. The grid was washed three times with PBS and allowed to semi-dry at room temperature before transmission electron microscopy observation (H7500; Hitachi, Tokyo, Japan).

### Mitochondrial Membrane Potential Measurements

Mitochondrial membrane potential (MMP) was determined using JC-1 (K1944, Bioswamp) according to the manufacture's instruction. Briefly, cells were incubated and washed with Invitrogen JC-1 solution according to the manufacturer's guidelines. Finally, all cells were harvested and analyzed by flow cytometry (NovoCyte; ACEA Biosciences, San Diego, CA). The data were analyzed using the built-in NovoCyte software.

### Assessment of Mitochondrial Permeability Transition Pore Opening

The mitochondrial permeability transition pore (mPTP) opening was examined using the Mitochondrial PT Pore Assay Kit (601430-100; Cayman Chemical, Ann Arbor, MI). Cells were incubated with 5 µL of calcein acetoxymethyl ester (Invitrogen) and 5 µL of CoCl_2_ for 15 minutes at 37°C. The calcein loaded into the mitochondria was preserved, and the cytosolic calcein was quenched by CoCl_2_. The results were analyzed using a NovoCyte flow cytometer. The data were analyzed using the built-in NovoCyte software.

### Transwell Assay

Cells were seeded at 1 × 10^5^ cells/mL (500 µL in each well) in the upper chamber of Transwell plates (3421; Corning, Corning, NY, USA) with serum-free medium, and medium containing 10% FBS was added to the lower chamber. Next, cells were transfected as described above for 48 h. The upper chamber was covered with cell culture medium and Matrigel (BD Biosciences, San Jose, CA) mixture. Finally, cells in the top chamber were removed with cotton swabs, while cells that went through the membrane were stained with 0.5% crystal violet. The cells were observed and counted under a microscope at 100× magnification.

### In Vitro HUVEC Tube Formation Assay

HUVECs were trypsinized and seeded in 96-well plates at 5 × 10^4^ cells per well into Matrigel (Corning) incubated for 4 hours. Following incubation, images of six randomly chosen fields in each well were taken. The number of neovascularizations and the number of nodes were calculated using ImageJ (National Institutes of Health, Bethesda, MA, USA).

### VEGF-A–Induced Mouse Corneal Neovascularization Model

The corneal neovascularization model^14^ was established in 25 healthy male BALB/c mice 7 to 8 weeks of age and weighing 20 ± 5 g. The mice were randomly divided into five groups (*n* = 5): model, shPXN, shEV, PXN, and empty vector. Control mice were not treated in any way. Briefly, the mice were anesthetized by intraperitoneal injection of sodium pentobarbital (40 mg/kg). VEGF-A was prepared in a 10-µg/100 µL PBS solution, and 200 ng of VEGF-A was injected subconjunctivally into the right eye of the mice for 7 consecutive days, once a day. Angiogenesis was observed using a slit lamp or under a microscope every 2 days. The left eye of the mice was used as a control. Lentiviral shPXN, shEV, PXN, or empty vector was injected subconjunctivally at 10 µg every 3 days for a total of 12 days (four injections). After treatment, the cornea was extracted under a slit lamp. The mice were sacrificed, and both eyeballs were immediately enucleated. Scleral corneal tissue (1-mm wide) was removed on ice; half was stored in at –80°C and the other half was fixed with 4% paraformaldehyde.

### Immunohistochemical Staining

The corneal tissues were fixed in 4% paraformaldehyde for 30 minutes, rinsed with PBS, and incubated in 0.1% Triton X-100 for 20 minutes at room temperature. After rinsing with PBS, the tissue sections were blocked with goat serum for 1 hour at 37°C. The sections were then rinsed with PBS and incubated with primary antibodies against HK1 (MAB37234, 1:50; Bioswamp), HK2 (PAB30271, 1:50; Bioswamp), and GLUT1 (MAB37348, 1:50; Bioswamp) overnight at 4°C. The sections were washed with PBS the next day and incubated with MaxVision secondary antibodies (KIT-5020; MXB Biotechnologies, Fuzhou, China) for 30 minutes at 37°C. The sections were then washed with PBS and mounted onto coverslips. Image analysis was performed using a Leica DM500 microscope (Leica Camera, Wetzlar, Germany).

### Statistical Analysis

One-way analysis of variance was performed to compare differences among multiple groups using SPSS Statistics 19.0 (IBM Corp., Armonk, NY, USA). The data are expressed as the mean ± standard deviation (SD). *P* < 0.05 was considered to be statistically significant.

## Results

### PXN Is Involved in the VEGF-A–Regulated Metabolism of HUVECs

**Figure 1.**
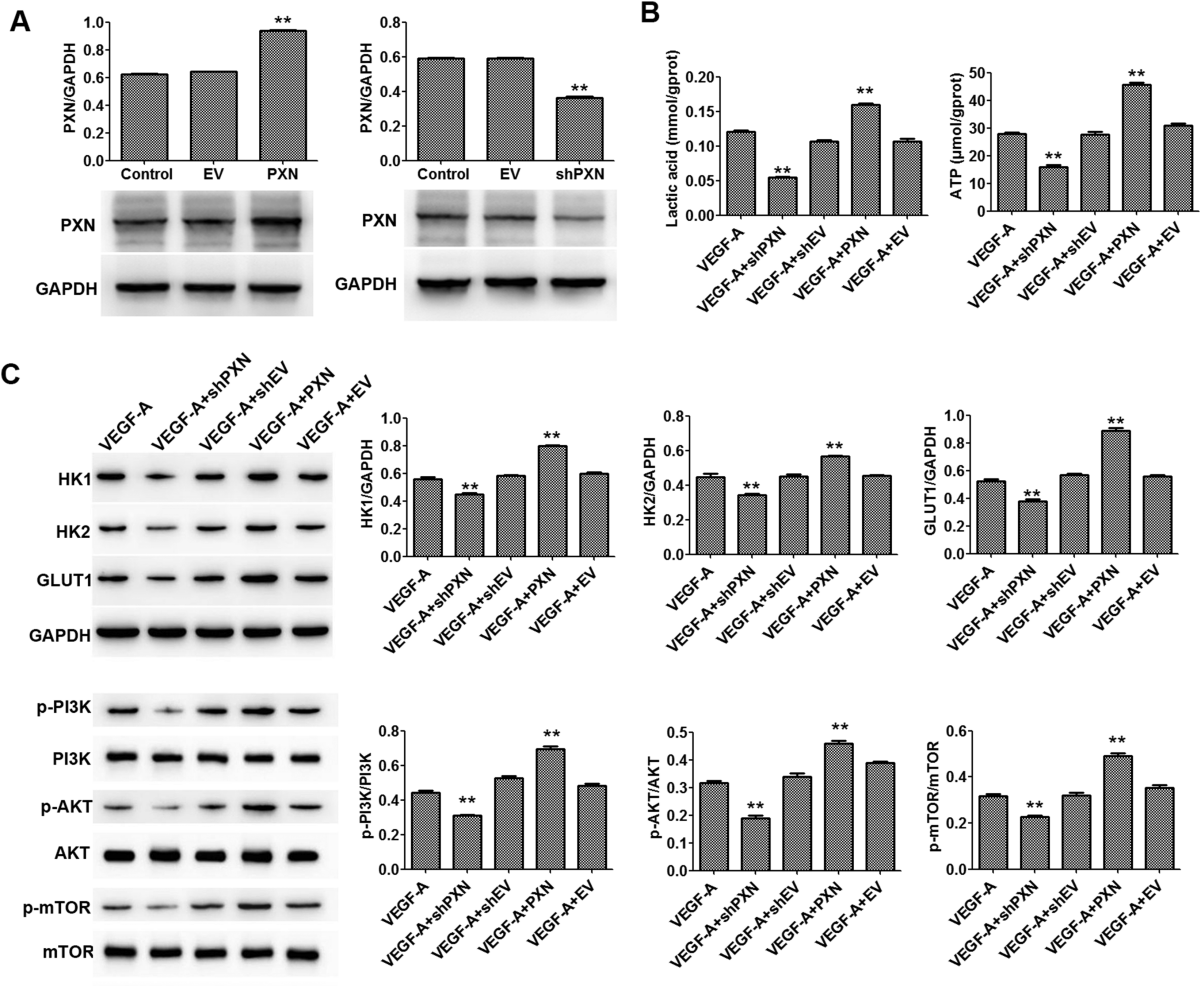
PXN interference inhibited the VEGF-A–induced metabolism of HUVECs. (**A**) Protein expression of PXN in HUVECs was detected by western blot. (**B**) Levels of lactic acid and ATP in HUVECs were detected by ELISA. (**C**) Protein expression levels of HK1, HK2, GLUT1, PI3K, phospho-PI3K, AKT, phospho-AKT, mTOR, and phospho-mTOR were detected by western blot. Data are shown as the mean ± SD (*n* = 3). ^**^P < 0.01 compared with control or VEGF-A.

To verify whether PXN is involved in the regulated metabolism of VEGF-A in HUVECS, we investigated the effects of overexpression and interference in lactate and ATP in HUVECs, as well as the changes in important proteins in the PI3K/AKT/mTOR signaling pathway. As shown in Figure 1A, PXN overexpression and interference significantly increased and decreased the protein levels of PXN in HUVECs, respectively. In turn, PXN interference and overexpression decreased and increased the levels of lactic acid and ATP in HUVECs, respectively (Fig. 1B). In addition, western blot showed that PXN interference and overexpression downregulated and upregulated the protein expression of HK1, HK2, and GLUT1 in HUVECs (Fig. 1C). We also found that PXN interference inhibited the activation of PI3K/AKT/mTOR signaling in HUVECs by reducing the phosphorylation of PI3K, AKT, and mTOR (Fig. 1C).

**Figure 2.**
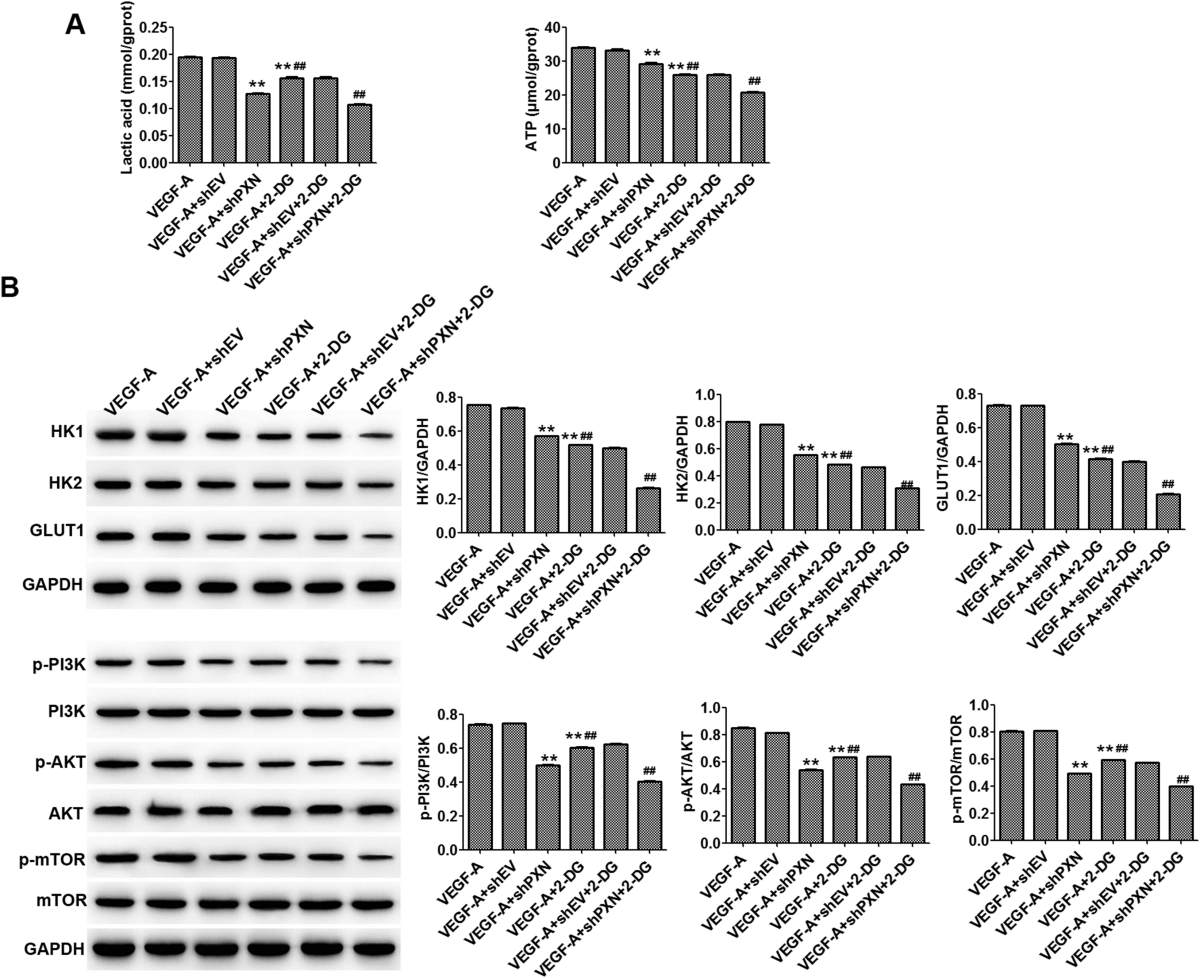
2-DG strengthens the inhibitory effect of PXN on HUVEC metabolism. (**A**) Levels of lactic acid and ATP in HUVECs were detected by ELISA. (**B**) Protein expression levels of HK1, HK2, GLUT1, PI3K, phospho-PI3K, AKT, phospho-AKT, mTOR, and phospho-mTOR were detected by western blot. Data are shown as the mean ± SD (*n* = 3). ^**^P < 0.01 compared with VEGF-A; ^##^*P* < 0.01 compared with VEGF-A + shPXN.

In order to further confirm that PXN is involved in regulating cell metabolism, we combined the glucose inhibitor 2-deoxy-d-glucose (2-DG) with PXN interference treatment in HUVECs. Compared with VEGF-A, PXN interference significantly decreased lactic acid and ATP production; downregulated HK1, HK2, and GLUT1; and promoted inactivation of PI3K/AKT/mTOR signaling. The same was observed when 2-DG was administered, and combined treatment with shPXN and 2-DG showed a stronger effect on the levels of ATP and lactic acid; the expression of HK1, HK2, and GLUT1; and the activation of PI3K/AKT/mTOR signaling in HUVECs compared with individual administration (Fig. 2).

### PXN Interference Alleviated VEGF-A–Induced Mitochondrial Damage in HUVECs

**Figure 3.**
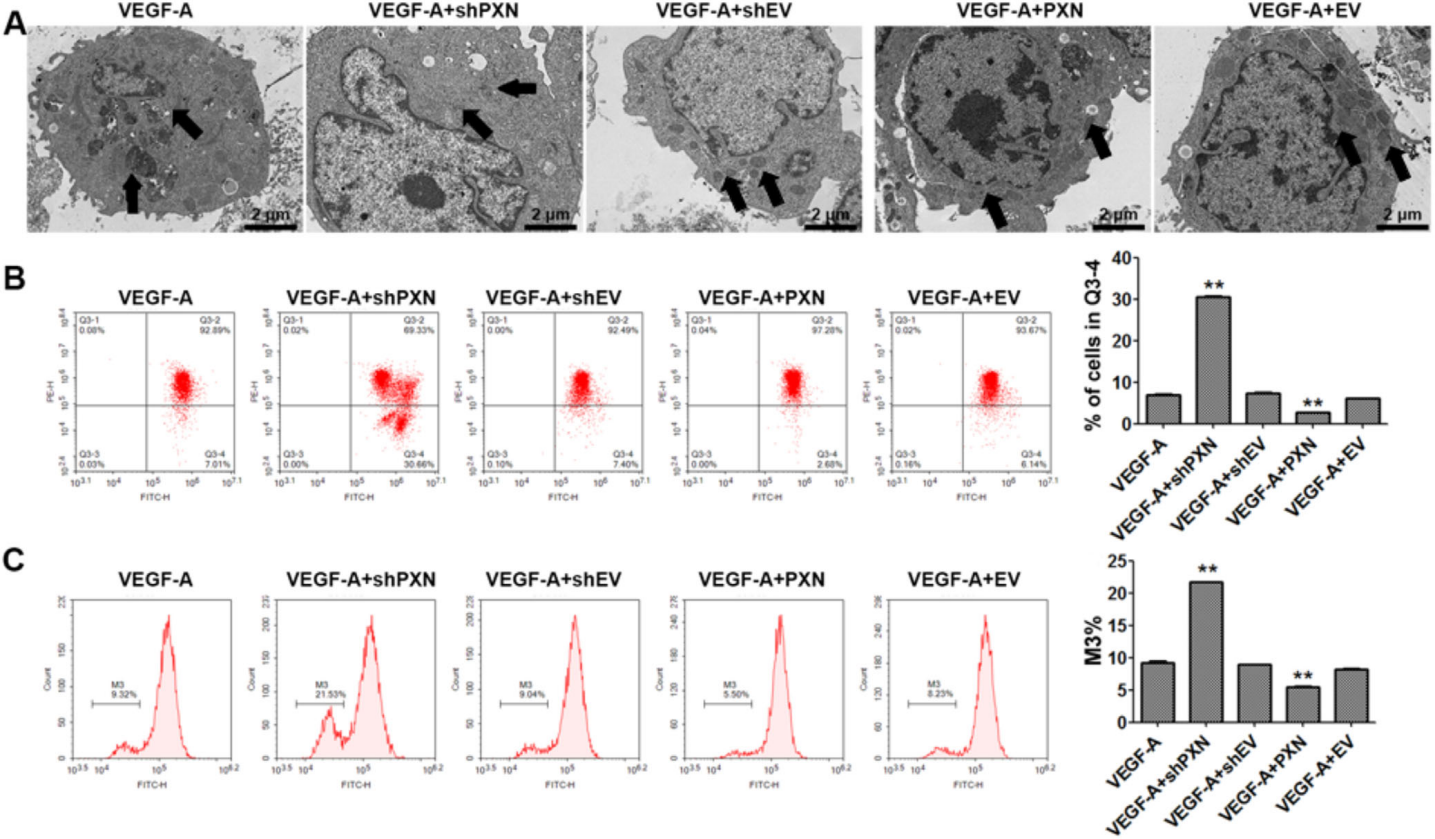
PXN interference alleviated VEGF-A–induced mitochondrial damage in HUVECs. (**A**) Transmission electron microscopy observation of mitochondrial morphology. *Scale bars:* 2 µm. (**B**) MMP in HUVECs was detected by JC-1 assay. (**C**) mPTP opening of HUVECs was detected by the CoCl_2_–calcein fluorescence-quenching assay. Data are shown as the mean ± SD (*n* = 3). ^**^P < 0.01 compared with control or VEGF-A.

PXN has been shown to affect cell migration via mitochondria; therefore, mitochondrial structure is critical in the process of vascular neogenesis.^3^ In VEGF-A–treated cells, the mitochondrial morphology was enlarged, and the outer and inner membranes of the mitochondria were barely distinguishable, but PXN interference significantly reversed these changes in mitochondria (Fig. 3A). Assays using fluorescent JC-1 probes demonstrated that shPXN decreased the MMP in HUVECs (Fig. 3B). We then measured the mPTP opening using the CoCl_2_–calcein fluorescence-quenching assay and found that the mPTP of PXN-silenced cells was increased significantly compared with that in VEGF-A–treated cells (Fig. 3C).

### miR-145-5p Inhibited the Metabolism, Invasion, and Angiogenesis of HUVECs Via Downregulation of PXN

**Figure 4.**
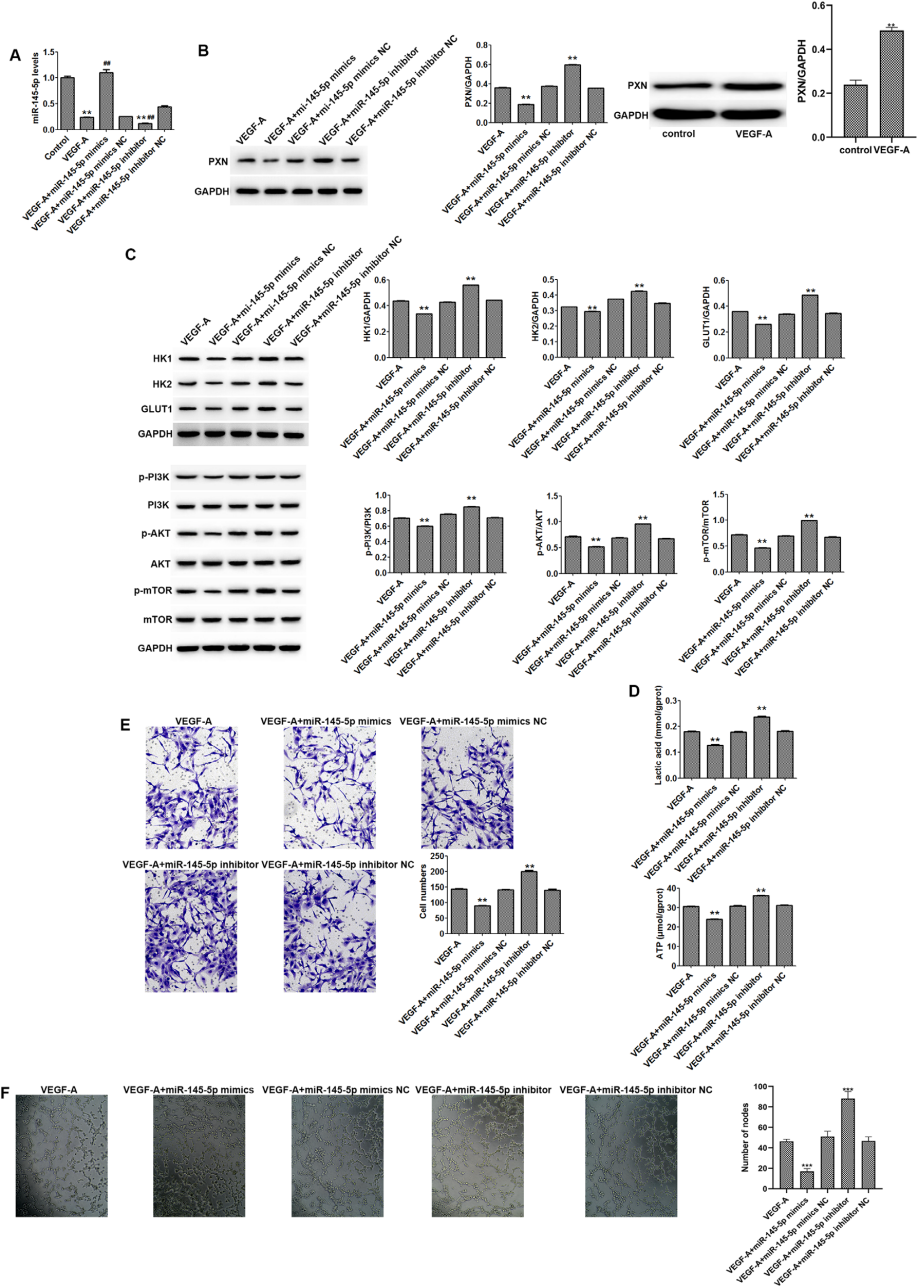
miR-145-5p inhibited HUVEC metabolism, invasion, and angiogenesis. (**A**) miR-145-5p expression in HUVECs was detected by quantitative RT-PCR. (**B**) Protein expression levels of PXN in HUVECs were detected by western blot. (**C**) Protein expression levels of HK1, HK2, GLUT1, PI3K, phospho-PI3K, AKT, phospho-AKT, mTOR, and phospho-mTOR were detected by western blot. (**D**) Levels of lactic acid and ATP in HUVECs were detected by ELISA. (**E**) HUVEC invasion was evaluated by Transwell assay (200× magnification). (**F**) miR-145-5p mimics inhibited the angiogenesis of HUVECs (200× magnification). Data are shown as the mean ± SD (*n* = 3). ^**^P < 0.01 compared with VEGF-A. NC, negative control.

miR-145 has been shown to inhibit tumor cell proliferation, migration, and invasion through the PI3K/AKT signaling pathway.^12^ To investigate whether miR-145 can influence the metabolism, invasion, and angiogenesis of HUVECs through the regulation of PNX, we investigated the changes of related factors. Compared with control cells, VEGF-A or miR-145-5p inhibitor significantly decreased the levels of miR-145-5p, and miR-145-5p mimics significantly upregulated the levels of miR-145-5p in HUVECs (Fig. 4A). miR-145-5p mimics and inhibitors decreased and increased the protein expression of PXN in HUVECs, respectively (Fig. 4B). In addition, miR-145-5p mimics decreased the protein levels of HK1, HK2, and GLUT1; inhibited the activation of PI3K/AKT/mTOR signaling; and reduced the levels of lactic acid and ATP in HUVECs (Figs. 4C, 4D). Furthermore, miR-145 mimics inhibited the invasion (Fig. 4E) and angiogenesis (Fig. 4F) of HUVECs.

### miR-145-5p Inhibitors Alleviated PXN-Inhibited Metabolism, Invasion, and Angiogenesis of HUVECs

**Figure 5.**
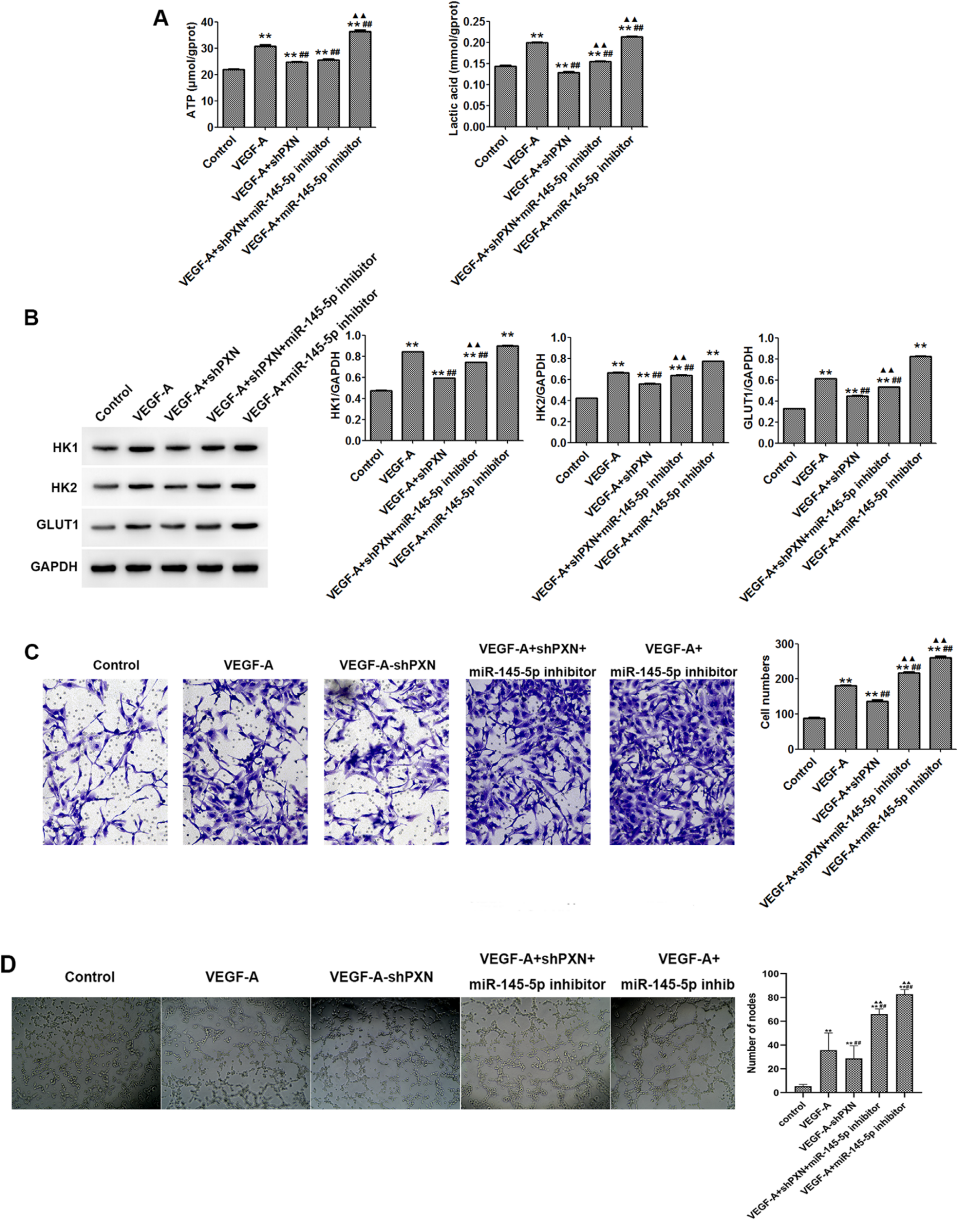
miR-145-5p inhibitors alleviated the PXN-inhibited metabolism, invasion, and angiogenesis of HUVECs. (**A**) Levels of lactic acid and ATP in HUVECs were detected by ELISA. (**B**) Protein expression levels of HK1, HK2, and GLUT1 were detected by western blot. (**C**) HUVEC invasion was evaluated by Transwell assay (200× magnification). (**D**) miR-145-5p inhibitors alleviated the PXN interference-inhibited angiogenesis of HUVECs (200× magnification). Data are shown as the mean ± SD (*n* = 3). ^**^P < 0.01 compared with control; ^##^*P* < 0.01 compared with VEGF-A; ^▲▲^*P* < 0.01 compared with VEGF-A + shPXN.

To further validate the role of miR-145HUVECs in cells, we obtained the following results. Compared with the VEGF-A treatment group, combined miR-145-5p inhibition and PXN interference significantly promoted ATP generation and lactic acid release (Fig. 5A); enhanced the protein expression of HK1, HK2, and GLUT1 (Fig. 5B); and induced the invasion and angiogenesis in HUVECs (Figs. 5C, 5D).

### PXN Interference Inhibited the Metabolism of Corneal Tissue in the Mouse Model

**Figure 6.**
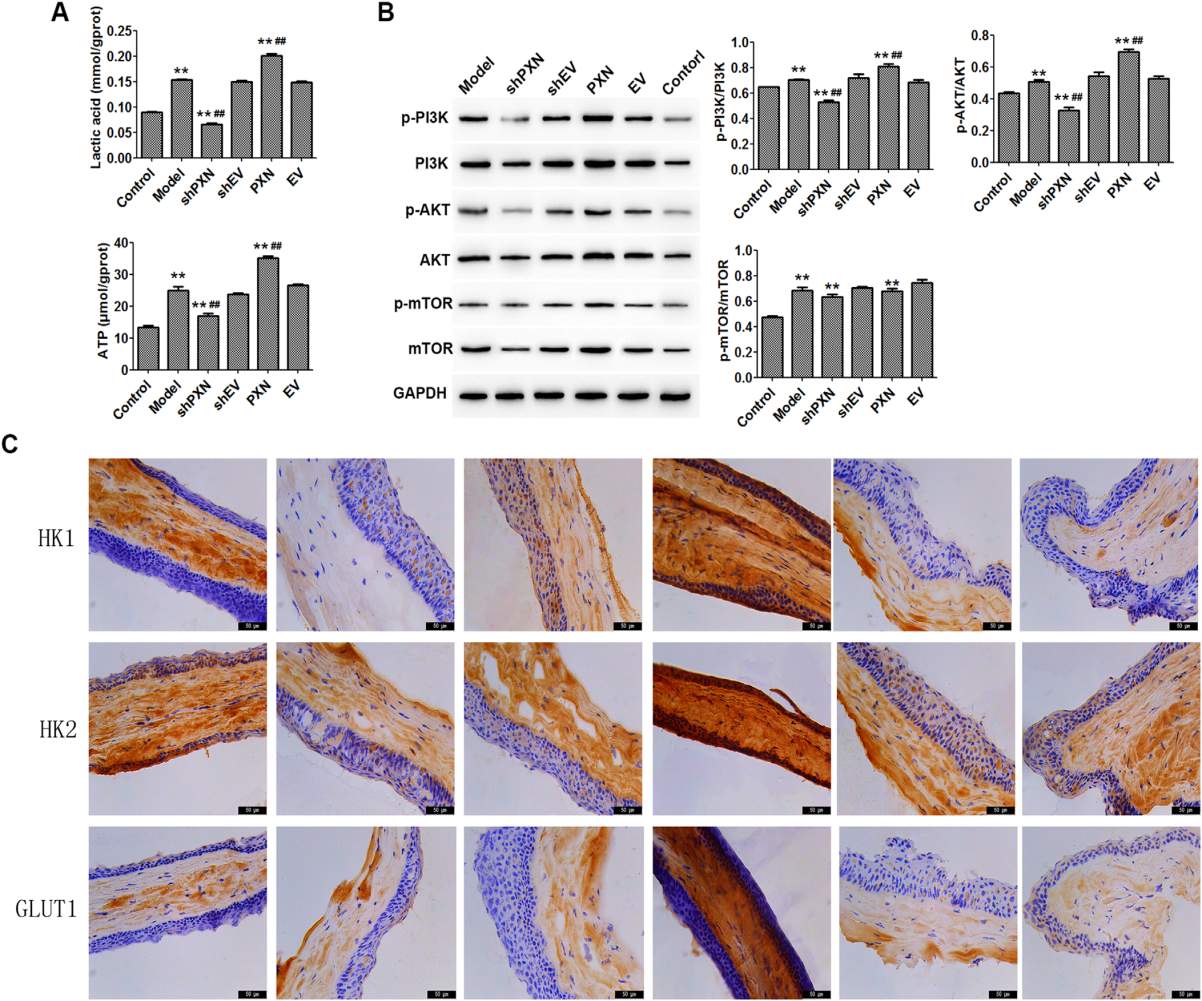
PXN interference inhibited mouse corneal tissue metabolism. (**A**) Levels of lactic acid and ATP in mouse corneal tissue were detected by ELISA. (**B**) Protein expression levels of PI3K, phospho-PI3K, AKT, phospho-AKT, mTOR, and phospho-mTOR were detected by western blot. (**C**) Levels of HK1, HK2, and GLUT1 in mouse corneal tissue were detected by immunohistochemical staining. *Scale* *bars:* 50 µm. Data are shown as the mean ± SD (*n* = 3). ^**^P < 0.01 compared with control; ^##^*P* < 0.01 compared with model.

The effect of PXN on the PI3K/AKT/mTOR signaling pathway has been studied previously. To investigate the specific effects of this pathway on corneal tissue, we detected the concentration of lactate and ATP and the expression of related proteins through PXN interference. As shown in Figures 6A and 6B, PXN interference significantly decreased the levels of lactic acid and ATP and inhibited the activation of PI3K/AKT/mTOR signaling in corneal tissues extracted from VEGF-A–treated mice. In addition, PXN interference significantly decreased the levels of HK1, HK2, and GLUT1 in mouse cornea (Fig. 6C).

## Discussion

Angiogenesis is an important process that occurs in healthy growth and wound healing, but it can also cause many malignant inflammatory diseases.^15^ Corneal neovascularization is a widespread condition associated with inflammation, infection, and trauma and can cause visual impairment, including blindness.^16^^,^^17^ In the process of angiogenesis, angiogenic growth factors stimulate endothelial cells to proliferate, migrate, and form tubular structures. VEGF-A plays an important role in promoting the permeability, proliferation, migration, and tube formation of endothelial cells and participates in the pathological process of various retinal diseases.^18^^–^^20^ In this study, we found that miR-145-5p–mediated downregulation of PXN significantly alleviated the VEGF-A–induced migration and angiogenesis of HUVECs by inhibiting mitochondrial energy metabolism via the PI3K/AKT/mTOR pathway.

Previous studies have demonstrated that VEGF-A promotes the activation of focal adhesions by inducing the phosphorylation of focal adhesion kinase (FAK) and its substrate PXN in HUVECs.^21^ Activation of FAK/PXN signaling induced angiogenesis in endothelial cells that is associated with the development of corneal neovascularization.^19^^,^^22^ PXN as a downstream signaling factor of FAK that can transduce adhesion and growth factor signals to regulate cell function. In the present study, PXN interference inhibited the migration and angiogenesis of HUVECs in the presence of VEGF-A. In addition, PXN interference decreased the production of lactic acid and ATP via downregulation of metabolism-related enzymes, reduced VEGF-A–induced mitochondrial damage, and inhibited PI3K/AKT/mTOR activation in HUVECs. The in vivo experimental results showed that PXN interference significantly reduced the VEGF-A–induced release of lactic acid and ATP, inhibited PI3K/AKT/mTOR activation, and decreased the levels of HK1, HK2, and GLUT1 in mouse corneal tissue. The results suggest that PXN mediates VEGF-A–induced angiogenesis in endothelial cells by regulating metabolism and mitochondrial damage via the PI3K/AKT/mTOR pathway.

In addition, we also found that the tumor suppressor miR-145-5p downregulated the VEGF-A–induced expression of PXN in HUVECs. Previous studies have revealed that the transfer of miR-145-5p from endothelial cell to neighboring colon cancer cells inhibited angiogenesis^23^ and that upregulation of miR-145-5p suppressed prostate cancer cell proliferation.^24^ In the present study, the overexpression of miR-145-5p in VEGF-A–induced HUVECs significantly decreased the protein expression of PXN and inhibited the invasion and angiogenesis of HUVECs in the presence of VEGF-A via regulation of PI3K/AKT/mTOR signaling. The results of the rescue experiment demonstrated that inhibiting the expression of miR-145-5p attenuated the protective effect of PXN interference on VEGF-A–induced HUVEC injury.

In conclusion, the present study indicated that PXN interference inhibited the VEGF-A–induced invasion and angiogenesis of HUVECs. Furthermore, PXN may be a target of miR-145-5p in regulating the angiogenesis in HUVECs via cell metabolism and mitochondrial damage. Therefore, PXN may be a potential target for antiangiogenic therapies.
